# Bis[(3-chloro­benz­yl)ammonium] 2-phenyl­propane­dioate dihydrate

**DOI:** 10.1107/S1600536810029764

**Published:** 2010-07-31

**Authors:** Jerry Joe Ebow Kingsley Harrison, Robert Kingsford-Adaboh, Kazuma Gotoh, Hiroyuki Ishida

**Affiliations:** a Department of Chemistry, Faculty of Science, University of Ghana, Box LG56 Legon, Accra, Ghana; b Department of Chemistry, Faculty of Science, Okayama University, Okayama 700-8530, Japan

## Abstract

In the asymmetric unit of the title compound, 2C_7_H_9_ClN^+^·C_9_H_6_O_4_^2−^·2H_2_O, there are two crystallographically independent cations, one dianion and two water mol­ecules. The dihedral angle between the two carboxyl­ate groups of the dianion is 78.1 (2)°. In the crystal, the components are held together by N—H⋯O, O—H⋯O and C—H⋯O hydrogen bonds, forming a layer parallel to the *bc* plane, with the hydro­philic and hydro­phobic groups located in the inner and outer regions of the layers, respectively.

## Related literature

For related structures, see: Ueda *et al.* (2005 ▶); Gotoh & Ishida (2009 ▶).
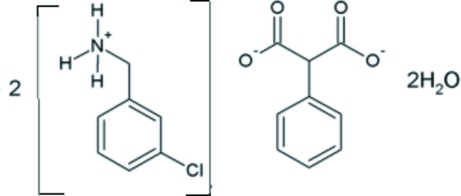


## Experimental

### 

#### Crystal data

2C_7_H_9_ClN^+^·C_9_H_6_O_4_^2−^·2H_2_O
*M* *_r_* = 499.39
Monoclinic, 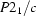

*a* = 17.3487 (7) Å
*b* = 9.7903 (5) Å
*c* = 14.3496 (6) Å
β = 103.3832 (12)°
*V* = 2371.07 (17) Å^3^
*Z* = 4
Mo *K*α radiation
μ = 0.32 mm^−1^
*T* = 93 K
0.36 × 0.25 × 0.10 mm

#### Data collection

Rigaku R-AXIS RAPID-II diffractometer
Absorption correction: multi-scan (*ABSCOR*; Higashi, 1995 ▶) *T*_min_ = 0.809, *T*_max_ = 0.969
22684 measured reflections
5404 independent reflections
4801 reflections with *I* > 2σ(*I*)
*R*_int_ = 0.026

#### Refinement

*R*[*F* ^2^ > 2σ(*F* ^2^)] = 0.030
*wR*(*F* ^2^) = 0.080
*S* = 1.03
5404 reflections
312 parameters
H atoms treated by a mixture of independent and constrained refinement
Δρ_max_ = 0.35 e Å^−3^
Δρ_min_ = −0.28 e Å^−3^

### 

Data collection: *PROCESS-AUTO* (Rigaku/MSC, 2004 ▶); cell refinement: *PROCESS-AUTO*; data reduction: *CrystalStructure* (Rigaku/MSC, 2004 ▶); program(s) used to solve structure: *SIR92* (Altomare *et al.*, 1994 ▶); program(s) used to refine structure: *SHELXL97* (Sheldrick, 2008 ▶); molecular graphics: *ORTEP-3* (Farrugia, 1997 ▶); software used to prepare material for publication: *CrystalStructure* (Rigaku/MSC, 2004 ▶).

## Supplementary Material

Crystal structure: contains datablocks global, I. DOI: 10.1107/S1600536810029764/zl2291sup1.cif

Structure factors: contains datablocks I. DOI: 10.1107/S1600536810029764/zl2291Isup2.hkl

Additional supplementary materials:  crystallographic information; 3D view; checkCIF report

## Figures and Tables

**Table 1:** Hydrogen-bond geometry (Å, °)

*D*—H⋯*A*	*D*—H	H⋯*A*	*D*⋯*A*	*D*—H⋯*A*
N1—H1*A*⋯O2	0.91	1.89	2.7789 (13)	165
N1—H1*B*⋯O1^i^	0.91	1.91	2.7891 (14)	163
N1—H1*C*⋯O4^ii^	0.91	1.82	2.7286 (13)	175
N2—H2*A*⋯O2	0.91	2.12	2.9783 (13)	157
N2—H2*A*⋯O4	0.91	2.40	2.9220 (13)	117
N2—H2*B*⋯O5^iii^	0.91	1.98	2.8530 (14)	159
N2—H2*C*⋯O5^i^	0.91	2.01	2.8863 (13)	162
O5—H5*A*⋯O1	0.795 (18)	1.931 (18)	2.7158 (12)	169.2 (16)
O5—H5*B*⋯O3^i^	0.841 (17)	1.868 (17)	2.6818 (12)	162.6 (15)
O6—H6*A*⋯O1	0.828 (17)	2.060 (17)	2.8559 (13)	161.0 (16)
O6—H6*B*⋯O3^iv^	0.856 (17)	1.945 (17)	2.7948 (13)	172.3 (16)
C9—H9⋯O4	0.95	2.58	3.2035 (15)	123
C15—H15⋯O2	0.95	2.39	3.2341 (15)	148
C22—H22⋯O3^v^	0.95	2.58	3.4936 (15)	160
C23—H23*A*⋯O6^vi^	0.99	2.49	3.3424 (15)	144

Symmetry codes: (i) 
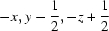
; (ii) 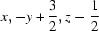; (iii) 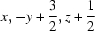; (iv) 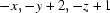; (v) 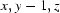; (vi) 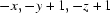.

**Fig. 1.**
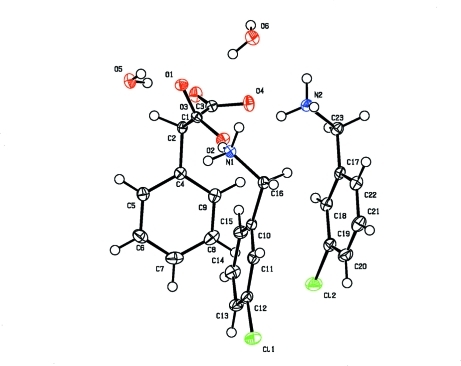
ORTEP-3 (Farrugia, 1997) diagram of 3-chlorobenzylammonium phenylmalonate dihydrate, showing the atom-numbering scheme. Displacement ellipsoids for non-hydrogen atoms are plotted at 50% probability and hydrogen atoms are shown as small spheres of arbitrary radii.

**Fig. 2.**
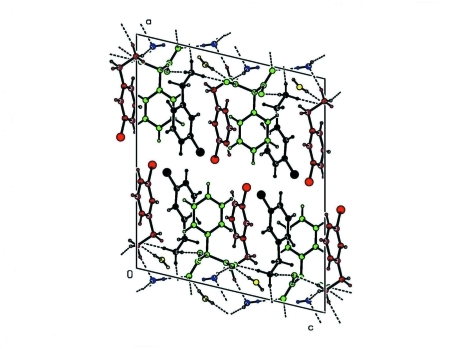
A part of the crystal packing of the crystal structure of 3-chlorobenzylammonium phenylmalonate dihydrate, viewed down the b axis showing a network of hydrogen bonds. Hydrogen bonds are shown by broken lines. Colour codes for residues: RED & BLACK = 3-chlorobenzylammonium; GREEN = phenylmalonate; BLUE & YELLOW = water molecules.

## supplementary crystallographic information

### Comment

The title compound was investigated as part of a structural study on *D*—H···*A* hydrogen bonding (*D* = N, O or C; *A* = N or O) in carboxylic acid and pyridine systems (Ueda *et al.*, 2005; Gotoh & Ishida, 2009).

The molecular structure of the compound and the crystal packing of the molecules viewed along the crystallographic *b* axis are shown in Figures 1 and 2, respectively. In its asymmetric unit the title compound has two crystallographically independent cations, one dianion and two water molecules. The two 3-chlorobenzylammonium cations have inverted but otherwise virtually indentical conformations as shown by the rotational angle of the ammonium group against the remainder of the molecule, N1-C16-C10-C11 = -119.04 (12)° and N2-C23-C17-C18 = 113.49 (12)° for the two groups (the two crystallographically independent ions are opposite enantiomers, but as as the structure is centrosymmetric both ions are present as racemic pairs throughout the structure).

The C—O bond length in one of the carboxylates is slightly longer than in the other with the respective values of O1—C1 and O2—C1 being 1.2714 (14) and 1.2501 (14) Å. The O3—C3 and O4—C3 bond lengths in the other carboxylates are 1.2559 (14) and 1.2538 (14) Å, respectively, making the carbonyl distance in the latter indistinguishable from the single bond due to resonance. The slight difference in the two sets of carbonyl distances may be attributed to the number of H bonds their respective O atoms are involved in, as H bonds tend to stabilize negative charge at the O atoms. The three O atoms with only two strong H bonds have C-O distances below 1.6 Å, the one with  three strong H bonds has a C-O distance larger than 1.7 Å.

The molecules associate by placement of all the phenyl rings in one direction, while the hydrophilic ammonium and the carboxylate ends are oriented towards the other end and are hydrogen bonded to water molecules, resulting in alternating hydrophobic and hydrophylic regions respectively in the crystal packing. In the hydrophilic regions the water molecules act as donors and acceptors in an extended hydrogen bonding network. Each water molecule serves as a bridge that links a 3-chlorobenzylammonium moiety and a phenylmalonate group. This arrangement affords an interconnectivity of water and donor protons such that two of the ammonium H atoms are donated to the water O atoms in a short N—H···O contact of comparable length [N2—H2B···O5^iii^ = 1.98Å and N2—H2C···O5^i^ = 2.01Å; Table 1], while the water H atoms are donated to the carboxylate O atoms [O5—H5A···O1 = 1.931 (18), O5—H5B···O3^i^ = 1.868 (17), O6—H6A···O1 = 2.060 (17) and O6—H6B···O3^iv^ = 1.945 (17) Å; Table 1].

In the hydrophobic regions the chloro-substituted aromatic rings show some stacking of parallel-shifted aromatic rings with each other, but the shortest centroid to centroid distance is larger than 4.3 Å and the interplanar separation is with greater than 3.6 Å also rather long for an attractive π-π stacking interaction. In the absence of any other directional interactions in the hydrophobic section of the structure it thus can be assumed that the crystal packing in these layers is likely to be dominated by shape recognition via dispersion forces.

### Experimental

Single crystals of the title compound were grown by slow evaporation of a water-ethanol solution (1:1 *v*/*v*; 20 ml) of 3-chlorobenzylamine (0.71 g, 5.0 mmol) and phenylmalonic acid (0.45 g, 0.25 mmol) at room temperature.

### Refinement

All H atoms were found in a difference Fourier map. Positional parameters of the water H atoms were refined, with *U*_iso_(H) = 1.2*U*_eq_(O). The NH_3_^+^ groups were refined as rigid groups (N—H = 0.91 Å), with *U*_iso_(H) = 1.5*U*_eq_(N), allowing for rotation around the C—N bonds. Other C-bound H atoms were refined as riding, with C—H = 0.95–1.00 Å, and with *U*_iso_(H) = 1.2*U*_eq_(C).

### Crystal data

**Table:**

2C_7_H_9_ClN^+^·C_9_H_6_O_4_^2^^−^·2H_2_O	*F*(000) = 1048.00
*M**_r_* = 499.39	*D*_x_ = 1.399 Mg m^−^^3^
Monoclinic, *P*2_1_/*c*	Mo *K*α radiation, λ = 0.71075 Å
Hall symbol: -P 2ybc	Cell parameters from 19587 reflections
*a* = 17.3487 (7) Å	θ = 3.2–27.5°
*b* = 9.7903 (5) Å	µ = 0.32 mm^−^^1^
*c* = 14.3496 (6) Å	*T* = 93 K
β = 103.3832 (12)°	Block, colorless
*V* = 2371.07 (17) Å^3^	0.36 × 0.25 × 0.10 mm
*Z* = 4	

### Data collection

**Table:**

Rigaku R-AXIS RAPID-II diffractometer	4801 reflections with *I* > 2σ(*I*)
Detector resolution: 10.00 pixels mm^-1^	*R*_int_ = 0.026
ω scans	θ_max_ = 27.5°, θ_min_ = 3.2°
Absorption correction: multi-scan (*ABSCOR*; Higashi, 1995)	*h* = −22→22
*T*_min_ = 0.809, *T*_max_ = 0.969	*k* = −12→12
22684 measured reflections	*l* = −17→18
5404 independent reflections	

### Refinement

**Table:**

Refinement on *F*^2^	312 parameters
Least-squares matrix: full	H atoms treated by a mixture of independent and constrained refinement
*R*[*F*^2^ > 2σ(*F*^2^)] = 0.030	*w* = 1/[σ^2^(*F*_o_^2^) + (0.045*P*)^2^ + 0.8144*P*] where *P* = (*F*_o_^2^ + 2*F*_c_^2^)/3
*wR*(*F*^2^) = 0.080	(Δ/σ)_max_ = 0.001
*S* = 1.03	Δρ_max_ = 0.35 e Å^−^^3^
5404 reflections	Δρ_min_ = −0.28 e Å^−^^3^

### Special details

**Table:**

Geometry. All e.s.d.'s (except the e.s.d. in the dihedral angle between two l.s. planes) are estimated using the full covariance matrix. The cell e.s.d.'s are taken into account individually in the estimation of e.s.d.'s in distances, angles and torsion angles; correlations between e.s.d.'s in cell parameters are only used when they are defined by crystal symmetry. An approximate (isotropic) treatment of cell e.s.d.'s is used for estimating e.s.d.'s involving l.s. planes.
Refinement. Refinement of *F*^2^ against ALL reflections. The weighted *R*-factor *wR* and goodness of fit *S* are based on *F*^2^, conventional *R*-factors *R* are based on *F*, with *F* set to zero for negative *F*^2^. The threshold expression of *F*^2^ > σ(*F*^2^) is used only for calculating *R*-factors(gt) *etc*. and is not relevant to the choice of reflections for refinement. *R*-factors based on *F*^2^ are statistically about twice as large as those based on *F*, and *R*- factors based on ALL data will be even larger.

### Fractional atomic coordinates and isotropic or equivalent isotropic displacement parameters (Å^2^)

**Table:**

	*x*	*y*	*z*	*U*_iso_*/*U*_eq_	
Cl1	0.439729 (18)	0.35812 (3)	0.16174 (2)	0.02351 (8)	
Cl2	0.456436 (18)	0.67256 (3)	0.58413 (2)	0.02689 (9)	
O1	0.01868 (5)	0.88588 (8)	0.27920 (6)	0.01579 (17)	
O2	0.12915 (5)	0.76812 (8)	0.33753 (6)	0.01682 (17)	
O3	0.10538 (5)	1.13611 (8)	0.50629 (6)	0.01916 (18)	
O4	0.11331 (5)	0.90909 (8)	0.52121 (6)	0.01844 (18)	
O5	−0.03654 (5)	0.85786 (9)	0.08701 (6)	0.01747 (17)	
H5A	−0.0194 (10)	0.8553 (16)	0.1434 (13)	0.021*	
H5B	−0.0583 (9)	0.7825 (18)	0.0693 (11)	0.021*	
O6	−0.06079 (6)	0.68283 (9)	0.36504 (7)	0.02034 (18)	
H6A	−0.0353 (10)	0.7262 (17)	0.3327 (12)	0.024*	
H6B	−0.0720 (9)	0.7440 (17)	0.4024 (12)	0.024*	
N1	0.10896 (6)	0.55243 (10)	0.20825 (7)	0.01391 (19)	
H1A	0.1134	0.6328	0.2409	0.021*	
H1B	0.0613	0.5135	0.2079	0.021*	
H1C	0.1126	0.5684	0.1469	0.021*	
N2	0.09606 (6)	0.61282 (10)	0.50244 (7)	0.0162 (2)	
H2A	0.1111	0.6780	0.4650	0.024*	
H2B	0.0510	0.6400	0.5193	0.024*	
H2C	0.0869	0.5329	0.4693	0.024*	
C1	0.09030 (7)	0.87682 (11)	0.32604 (7)	0.0126 (2)	
C2	0.12851 (6)	1.00848 (11)	0.37329 (8)	0.0127 (2)	
H2	0.1016	1.0877	0.3352	0.015*	
C3	0.11362 (6)	1.01818 (11)	0.47553 (8)	0.0136 (2)	
C4	0.21663 (7)	1.01807 (11)	0.37713 (8)	0.0138 (2)	
C5	0.24195 (7)	1.08640 (12)	0.30411 (8)	0.0173 (2)	
H5	0.2040	1.1274	0.2534	0.021*	
C6	0.32227 (8)	1.09505 (13)	0.30482 (10)	0.0225 (3)	
H6	0.3387	1.1402	0.2541	0.027*	
C7	0.37820 (7)	1.03784 (13)	0.37942 (10)	0.0238 (3)	
H7	0.4330	1.0452	0.3806	0.029*	
C8	0.35360 (7)	0.96960 (13)	0.45254 (9)	0.0227 (3)	
H8	0.3918	0.9301	0.5037	0.027*	
C9	0.27344 (7)	0.95889 (12)	0.45108 (9)	0.0188 (2)	
H9	0.2572	0.9109	0.5008	0.023*	
C10	0.25586 (7)	0.50774 (12)	0.25364 (8)	0.0152 (2)	
C11	0.30228 (7)	0.42829 (12)	0.20729 (8)	0.0170 (2)	
H11	0.2804	0.3502	0.1715	0.020*	
C12	0.38073 (7)	0.46418 (12)	0.21377 (8)	0.0171 (2)	
C13	0.41351 (7)	0.58019 (12)	0.26201 (9)	0.0195 (2)	
H13	0.4672	0.6038	0.2655	0.023*	
C14	0.36614 (7)	0.66165 (13)	0.30541 (9)	0.0215 (3)	
H14	0.3874	0.7430	0.3374	0.026*	
C15	0.28821 (7)	0.62547 (12)	0.30251 (9)	0.0188 (2)	
H15	0.2569	0.6808	0.3338	0.023*	
C16	0.17387 (7)	0.45831 (12)	0.25573 (9)	0.0175 (2)	
H16A	0.1651	0.3681	0.2238	0.021*	
H16B	0.1710	0.4456	0.3233	0.021*	
C17	0.23541 (7)	0.54046 (12)	0.56655 (8)	0.0163 (2)	
C18	0.30291 (7)	0.62186 (12)	0.58334 (8)	0.0175 (2)	
H18	0.3022	0.7109	0.6094	0.021*	
C19	0.37141 (7)	0.57119 (13)	0.56150 (9)	0.0197 (2)	
C20	0.37432 (8)	0.44271 (13)	0.52259 (9)	0.0227 (3)	
H20	0.4216	0.4105	0.5074	0.027*	
C21	0.30677 (8)	0.36155 (13)	0.50613 (9)	0.0227 (3)	
H21	0.3077	0.2730	0.4794	0.027*	
C22	0.23794 (7)	0.40932 (12)	0.52858 (9)	0.0191 (2)	
H22	0.1923	0.3527	0.5181	0.023*	
C23	0.16042 (7)	0.59252 (12)	0.59052 (8)	0.0176 (2)	
H23A	0.1425	0.5264	0.6332	0.021*	
H23B	0.1715	0.6803	0.6253	0.021*	

### Atomic displacement parameters (Å^2^)

**Table:**

	*U*^11^	*U*^22^	*U*^33^	*U*^12^	*U*^13^	*U*^23^
Cl1	0.01977 (15)	0.02374 (15)	0.02822 (16)	0.00445 (11)	0.00803 (12)	−0.00380 (12)
Cl2	0.01780 (15)	0.02676 (17)	0.03560 (18)	−0.00173 (11)	0.00518 (13)	0.00059 (13)
O1	0.0139 (4)	0.0185 (4)	0.0145 (4)	0.0003 (3)	0.0021 (3)	−0.0018 (3)
O2	0.0175 (4)	0.0127 (4)	0.0199 (4)	0.0010 (3)	0.0035 (3)	−0.0034 (3)
O3	0.0283 (5)	0.0130 (4)	0.0172 (4)	0.0028 (3)	0.0073 (4)	−0.0026 (3)
O4	0.0290 (5)	0.0124 (4)	0.0146 (4)	−0.0008 (3)	0.0065 (3)	0.0003 (3)
O5	0.0221 (4)	0.0164 (4)	0.0127 (4)	−0.0040 (3)	0.0017 (3)	−0.0005 (3)
O6	0.0251 (5)	0.0176 (4)	0.0199 (4)	−0.0012 (3)	0.0084 (4)	−0.0022 (3)
N1	0.0148 (4)	0.0134 (4)	0.0135 (4)	−0.0012 (4)	0.0031 (4)	−0.0012 (3)
N2	0.0158 (5)	0.0133 (5)	0.0197 (5)	0.0012 (4)	0.0043 (4)	0.0015 (4)
C1	0.0146 (5)	0.0151 (5)	0.0088 (5)	−0.0007 (4)	0.0044 (4)	−0.0011 (4)
C2	0.0157 (5)	0.0101 (5)	0.0119 (5)	0.0011 (4)	0.0027 (4)	0.0003 (4)
C3	0.0136 (5)	0.0139 (5)	0.0129 (5)	−0.0004 (4)	0.0020 (4)	−0.0017 (4)
C4	0.0162 (5)	0.0100 (5)	0.0149 (5)	−0.0005 (4)	0.0028 (4)	−0.0031 (4)
C5	0.0200 (6)	0.0140 (5)	0.0180 (5)	−0.0005 (4)	0.0045 (5)	−0.0008 (4)
C6	0.0238 (6)	0.0185 (6)	0.0286 (6)	−0.0024 (5)	0.0127 (5)	−0.0013 (5)
C7	0.0167 (6)	0.0196 (6)	0.0356 (7)	−0.0015 (5)	0.0074 (5)	−0.0074 (5)
C8	0.0193 (6)	0.0204 (6)	0.0252 (6)	0.0036 (5)	−0.0016 (5)	−0.0021 (5)
C9	0.0204 (6)	0.0176 (6)	0.0174 (5)	0.0006 (4)	0.0022 (5)	0.0005 (4)
C10	0.0147 (5)	0.0146 (5)	0.0153 (5)	0.0004 (4)	0.0012 (4)	0.0030 (4)
C11	0.0182 (5)	0.0143 (5)	0.0177 (5)	−0.0007 (4)	0.0022 (5)	−0.0003 (4)
C12	0.0172 (5)	0.0162 (5)	0.0177 (5)	0.0036 (4)	0.0038 (4)	0.0015 (4)
C13	0.0140 (5)	0.0191 (6)	0.0247 (6)	−0.0015 (4)	0.0028 (5)	0.0010 (5)
C14	0.0200 (6)	0.0166 (6)	0.0265 (6)	−0.0024 (4)	0.0027 (5)	−0.0042 (5)
C15	0.0179 (6)	0.0178 (6)	0.0208 (6)	0.0010 (4)	0.0046 (5)	−0.0022 (4)
C16	0.0159 (5)	0.0152 (5)	0.0210 (6)	−0.0002 (4)	0.0036 (5)	0.0039 (4)
C17	0.0181 (5)	0.0166 (5)	0.0134 (5)	0.0041 (4)	0.0023 (4)	0.0027 (4)
C18	0.0197 (6)	0.0154 (5)	0.0168 (5)	0.0024 (4)	0.0028 (5)	0.0009 (4)
C19	0.0174 (5)	0.0212 (6)	0.0192 (6)	0.0007 (5)	0.0014 (5)	0.0040 (5)
C20	0.0201 (6)	0.0243 (6)	0.0237 (6)	0.0072 (5)	0.0053 (5)	0.0009 (5)
C21	0.0262 (6)	0.0172 (6)	0.0231 (6)	0.0052 (5)	0.0025 (5)	−0.0024 (5)
C22	0.0194 (6)	0.0165 (5)	0.0196 (6)	0.0007 (4)	0.0007 (5)	0.0003 (4)
C23	0.0182 (5)	0.0175 (5)	0.0165 (5)	0.0018 (4)	0.0031 (5)	−0.0006 (4)

### Geometric parameters (Å, °)

**Table:**

Cl1—C12	1.7427 (12)	C8—C9	1.3899 (17)
Cl2—C19	1.7448 (13)	C8—H8	0.9500
O1—C1	1.2714 (14)	C9—H9	0.9500
O2—C1	1.2501 (14)	C10—C11	1.3941 (16)
O3—C3	1.2559 (14)	C10—C15	1.3975 (16)
O4—C3	1.2538 (14)	C10—C16	1.5095 (16)
O5—H5A	0.795 (18)	C11—C12	1.3878 (16)
O5—H5B	0.840 (18)	C11—H11	0.9500
O6—H6A	0.828 (18)	C12—C13	1.3822 (17)
O6—H6B	0.855 (17)	C13—C14	1.3914 (18)
N1—C16	1.4921 (14)	C13—H13	0.9500
N1—H1A	0.9100	C14—C15	1.3888 (17)
N1—H1B	0.9100	C14—H14	0.9500
N1—H1C	0.9100	C15—H15	0.9500
N2—C23	1.4935 (15)	C16—H16A	0.9900
N2—H2A	0.9100	C16—H16B	0.9900
N2—H2B	0.9100	C17—C18	1.3906 (17)
N2—H2C	0.9100	C17—C22	1.3994 (16)
C1—C2	1.5338 (15)	C17—C23	1.5092 (16)
C2—C4	1.5199 (15)	C18—C19	1.3889 (17)
C2—C3	1.5503 (15)	C18—H18	0.9500
C2—H2	1.0000	C19—C20	1.3818 (18)
C4—C9	1.3960 (16)	C20—C21	1.3901 (19)
C4—C5	1.3969 (16)	C20—H20	0.9500
C5—C6	1.3937 (17)	C21—C22	1.3880 (18)
C5—H5	0.9500	C21—H21	0.9500
C6—C7	1.3858 (19)	C22—H22	0.9500
C6—H6	0.9500	C23—H23A	0.9900
C7—C8	1.3916 (19)	C23—H23B	0.9900
C7—H7	0.9500		
			
H5A—O5—H5B	108.4 (15)	C15—C10—C16	121.68 (11)
H6A—O6—H6B	102.7 (15)	C12—C11—C10	119.51 (11)
C16—N1—H1A	109.5	C12—C11—H11	120.2
C16—N1—H1B	109.5	C10—C11—H11	120.2
H1A—N1—H1B	109.5	C13—C12—C11	121.67 (11)
C16—N1—H1C	109.5	C13—C12—Cl1	119.33 (9)
H1A—N1—H1C	109.5	C11—C12—Cl1	118.99 (9)
H1B—N1—H1C	109.5	C12—C13—C14	118.56 (11)
C23—N2—H2A	109.5	C12—C13—H13	120.7
C23—N2—H2B	109.5	C14—C13—H13	120.7
H2A—N2—H2B	109.5	C15—C14—C13	120.81 (11)
C23—N2—H2C	109.5	C15—C14—H14	119.6
H2A—N2—H2C	109.5	C13—C14—H14	119.6
H2B—N2—H2C	109.5	C14—C15—C10	120.02 (11)
O2—C1—O1	124.07 (10)	C14—C15—H15	120.0
O2—C1—C2	119.50 (10)	C10—C15—H15	120.0
O1—C1—C2	116.40 (9)	N1—C16—C10	114.08 (9)
C4—C2—C1	113.34 (9)	N1—C16—H16A	108.7
C4—C2—C3	110.39 (9)	C10—C16—H16A	108.7
C1—C2—C3	108.67 (9)	N1—C16—H16B	108.7
C4—C2—H2	108.1	C10—C16—H16B	108.7
C1—C2—H2	108.1	H16A—C16—H16B	107.6
C3—C2—H2	108.1	C18—C17—C22	119.48 (11)
O4—C3—O3	125.78 (10)	C18—C17—C23	120.18 (11)
O4—C3—C2	117.67 (9)	C22—C17—C23	120.33 (11)
O3—C3—C2	116.51 (10)	C19—C18—C17	119.10 (11)
C9—C4—C5	118.66 (11)	C19—C18—H18	120.4
C9—C4—C2	121.95 (10)	C17—C18—H18	120.4
C5—C4—C2	119.39 (10)	C20—C19—C18	121.97 (12)
C6—C5—C4	120.74 (11)	C20—C19—Cl2	118.99 (10)
C6—C5—H5	119.6	C18—C19—Cl2	119.04 (10)
C4—C5—H5	119.6	C19—C20—C21	118.74 (11)
C7—C6—C5	120.08 (12)	C19—C20—H20	120.6
C7—C6—H6	120.0	C21—C20—H20	120.6
C5—C6—H6	120.0	C22—C21—C20	120.31 (12)
C6—C7—C8	119.63 (12)	C22—C21—H21	119.8
C6—C7—H7	120.2	C20—C21—H21	119.8
C8—C7—H7	120.2	C21—C22—C17	120.38 (11)
C9—C8—C7	120.34 (12)	C21—C22—H22	119.8
C9—C8—H8	119.8	C17—C22—H22	119.8
C7—C8—H8	119.8	N2—C23—C17	111.54 (9)
C8—C9—C4	120.53 (11)	N2—C23—H23A	109.3
C8—C9—H9	119.7	C17—C23—H23A	109.3
C4—C9—H9	119.7	N2—C23—H23B	109.3
C11—C10—C15	119.37 (11)	C17—C23—H23B	109.3
C11—C10—C16	118.79 (10)	H23A—C23—H23B	108.0
			
O2—C1—C2—C4	35.03 (14)	C10—C11—C12—C13	−2.61 (18)
O1—C1—C2—C4	−147.07 (10)	C10—C11—C12—Cl1	176.06 (9)
O2—C1—C2—C3	−88.10 (12)	C11—C12—C13—C14	0.44 (18)
O1—C1—C2—C3	89.81 (11)	Cl1—C12—C13—C14	−178.22 (10)
C4—C2—C3—O4	−89.86 (12)	C12—C13—C14—C15	1.64 (19)
C1—C2—C3—O4	35.02 (13)	C13—C14—C15—C10	−1.55 (19)
C4—C2—C3—O3	87.97 (12)	C11—C10—C15—C14	−0.64 (18)
C1—C2—C3—O3	−147.15 (10)	C16—C10—C15—C14	174.74 (11)
C1—C2—C4—C9	−85.18 (13)	C11—C10—C16—N1	−119.05 (12)
C3—C2—C4—C9	36.99 (14)	C15—C10—C16—N1	65.55 (14)
C1—C2—C4—C5	93.96 (12)	C22—C17—C18—C19	0.32 (17)
C3—C2—C4—C5	−143.87 (10)	C23—C17—C18—C19	179.41 (10)
C9—C4—C5—C6	0.20 (17)	C17—C18—C19—C20	0.70 (18)
C2—C4—C5—C6	−178.97 (10)	C17—C18—C19—Cl2	−179.13 (9)
C4—C5—C6—C7	−1.26 (18)	C18—C19—C20—C21	−0.84 (19)
C5—C6—C7—C8	1.21 (19)	Cl2—C19—C20—C21	178.99 (10)
C6—C7—C8—C9	−0.12 (19)	C19—C20—C21—C22	−0.04 (19)
C7—C8—C9—C4	−0.95 (18)	C20—C21—C22—C17	1.05 (19)
C5—C4—C9—C8	0.90 (17)	C18—C17—C22—C21	−1.18 (18)
C2—C4—C9—C8	−179.95 (11)	C23—C17—C22—C21	179.73 (11)
C15—C10—C11—C12	2.67 (17)	C18—C17—C23—N2	113.48 (12)
C16—C10—C11—C12	−172.85 (10)	C22—C17—C23—N2	−67.43 (14)

### Hydrogen-bond geometry (Å, °)

**Table:**

*D*—H···*A*	*D*—H	H···*A*	*D*···*A*	*D*—H···*A*
N1—H1A···O2	0.91	1.89	2.7789 (13)	165
N1—H1B···O1^i^	0.91	1.91	2.7891 (14)	163
N1—H1C···O4^ii^	0.91	1.82	2.7286 (13)	175
N2—H2A···O2	0.91	2.12	2.9783 (13)	157
N2—H2A···O4	0.91	2.40	2.9220 (13)	117
N2—H2B···O5^iii^	0.91	1.98	2.8530 (14)	159
N2—H2C···O5^i^	0.91	2.01	2.8863 (13)	162
O5—H5A···O1	0.795 (18)	1.931 (18)	2.7158 (12)	169.2 (16)
O5—H5B···O3^i^	0.841 (17)	1.868 (17)	2.6818 (12)	162.6 (15)
O6—H6A···O1	0.828 (17)	2.060 (17)	2.8559 (13)	161.0 (16)
O6—H6B···O3^iv^	0.856 (17)	1.945 (17)	2.7948 (13)	172.3 (16)
C9—H9···O4	0.95	2.58	3.2035 (15)	123
C15—H15···O2	0.95	2.39	3.2341 (15)	148
C22—H22···O3^v^	0.95	2.58	3.4936 (15)	160
C23—H23A···O6^vi^	0.99	2.49	3.3424 (15)	144

Symmetry codes: (i) −*x*, *y*−1/2, −*z*+1/2; (ii) *x*, −*y*+3/2, *z*−1/2; (iii) *x*, −*y*+3/2, *z*+1/2; (iv) −*x*, −*y*+2, −*z*+1; (v) *x*, *y*−1, *z*; (vi) −*x*, −*y*+1, −*z*+1.
